# Inhibition of cathepsin proteases attenuates migration and sensitizes aggressive N-Myc amplified human neuroblastoma cells to doxorubicin

**DOI:** 10.18632/oncotarget.3579

**Published:** 2015-03-14

**Authors:** Lahiru Gangoda, Shivakumar Keerthikumar, Pamali Fonseka, Laura E. Edgington, Ching-Seng Ang, Cemil Ozcitti, Matthew Bogyo, Belinda S. Parker, Suresh Mathivanan

**Affiliations:** ^1^ Department of Biochemistry, La Trobe Institute for Molecular Science, La Trobe University, Bundoora, Victoria, Australia; ^2^ Bio21 Institute, University of Melbourne, Victoria, Australia; ^3^ Departments of Pathology and Microbiology and Immunology, Stanford University, Stanford, California, USA

**Keywords:** Secretome, proteomics, mass spectrometry, neuroblastoma, N-Myc amplification

## Abstract

Neuroblastoma arises from the sympathetic nervous system and accounts for 15% of childhood cancer mortality. Amplification of the oncogene N-Myc is reported to occur in more than 20% of patients. While N-Myc amplification status strongly correlates with higher tumour aggression and resistance to treatment, the role of N-Myc in the aggressive progression of the disease is poorly understood. N-Myc being a transcription factor can modulate the secretion of key proteins that may play a pivotal role in tumorigenesis. Characterising the soluble secreted proteins or secretome will aid in understanding their role in the tumour microenvironment, such as promoting cancer cell invasion and resistance to treatment. The aim of this study is to characterise the secretome of human malignant neuroblastoma SK-N-BE2 (N-Myc amplified, more aggressive) and SH-SY5Y (N-Myc non-amplified, less aggressive) cells. Conditioned media from SK-N-BE2 and SH-SY5Y cell lines were subjected to proteomics analysis. We report a catalogue of 894 proteins identified in the secretome isolated from the two neuroblastoma cell lines, SK-N-BE2 and SH-SY5Y. Functional enrichment analysis using FunRich software identified enhanced secretion of proteins implicated in cysteine peptidase activity in the aggressive N-Myc amplified SK-N-BE2 secretome compared to the less tumorigenic SH-SY5Y cells. Protein-protein interaction-based network analysis highlighted the enrichment of cathepsin and epithelial-to-mesenchymal transition sub-networks. For the first time, inhibition of cathepsins by inhibitors sensitized the resistant SK-N-BE2 cells to doxorubicin as well as decreased its migratory potential. The dataset of secretome proteins of N-Myc amplified (more aggressive) and non-amplified (less aggressive) neuroblastoma cells represent the first inventory of neuroblastoma secretome. The study also highlights the prominent role of cathepsins in the N-Myc amplified neuroblastoma pathogenesis. As N-Myc amplification correlates with aggressive neuroblastoma and chemotherapy-based treatment failure, co-treatment with cathepsin inhibitors might be a better avenue for disease management.

## INTRODUCTION

Cancer development is a multistep process in which somatic cells accumulate genetic modifications as a result of an initial environmental insult followed by a promoting event [1]. For the cancer to progress, bidirectional cross-talk between different cells within the tumour and its surrounding supporting tissue or tumour stroma is pivotal [2]. Stromal elements include the extracellular matrix (ECM), as well as other cell types that are activated and/or recruited to the tumour microenvironment such as fibroblasts, adipocytes, endothelial cells, and immune cell infiltrates [3]. It is well established that many aspects of cellular tumourigenicity are profoundly influenced by reciprocal interactions between the responding normal cells, their mediators, structural components of the ECM, and genetically altered neoplastic cells [4-7].

Within the cellular microenvironment, secreted proteins play a direct role in the regulation of both physiological and pathophysiological processes [8-10]. These secreted proteins include growth factors, ECM-degrading enzymes, cell motility factors, angiogenic factors and immunoregulatory cytokines participating in various physiological processes such as immune defense and cell signaling [11]. Such bioactive molecules also play critical roles in pathological processes including cancer cell differentiation, invasion, metastasis and sustained angiogenesis by regulating cell-cell adhesion and ECM interactions [12]. Furthermore, proteins secreted by cancer cells into the tumour microenvironment (referred to as cancer secretome) can subsequently enter bodily fluids, such as blood and urine, and can be exploited as disease biomarkers for diagnostic and prognostic purposes [13]. Hence, a detailed characterisation of secreted proteins and their critical role in cell physiology and signalling pathways may increase understanding of the disease and facilitate development of improved therapies.

Neuroblastoma is the most common extracranial solid tumor in childhood [14]. Despite aggressive treatment strategies, the prognosis of patients with disseminated neuroblastoma is grim, with a 5-year survival rate of approximately 30%. The disease initiates from primitive neuroepithelial cells of the embryonic neural crest that develop into tumours within the sympathetic nervous system [14]. Amplification of the oncogene N-Myc is detected in approximately 20% of patients, and >10 copies is strongly correlated with higher tumour aggression and resistance to treatment. Notably, the presence of the N-Myc oncogene is generally an indicator for high-risk due to treatment resistance [15]. While the degree of malignancy, prognosis and resistance to therapy is associated with the amplification of N-Myc, its expression levels correlate with tumour growth and invasiveness [16, 17]. By regulating gene expression through its activity as a transcription factor, N-Myc plays a key role in signalling cascades involved in cell proliferation such as PI3K/AKT/mTOR or MAPK/ERK [18]. Moreover, N-Myc amplification has been associated with increased levels of vascular endothelial growth factor (VEGF) thereby promoting angiogenesis in neuroblastoma [19].

To date, very little is known about the soluble secreted proteins that could modulate the aggressive phenotype of N-Myc amplified neuroblastoma cells. To investigate this, we profiled the cancer secretome of N-Myc amplified and non-amplified neuroblastoma cells by high resolution mass spectrometry-based label-free quantitative proteomics analysis. Bioinformatics analyses of the secreted proteins were carried out to short list potential N-Myc targets with relevance to neuroblastoma pathogenesis.

## RESULTS AND DISCUSSION

### Proteomics analysis of SH-SY5Y and SK-N-BE2 cell line secretome

**Figure 1:**
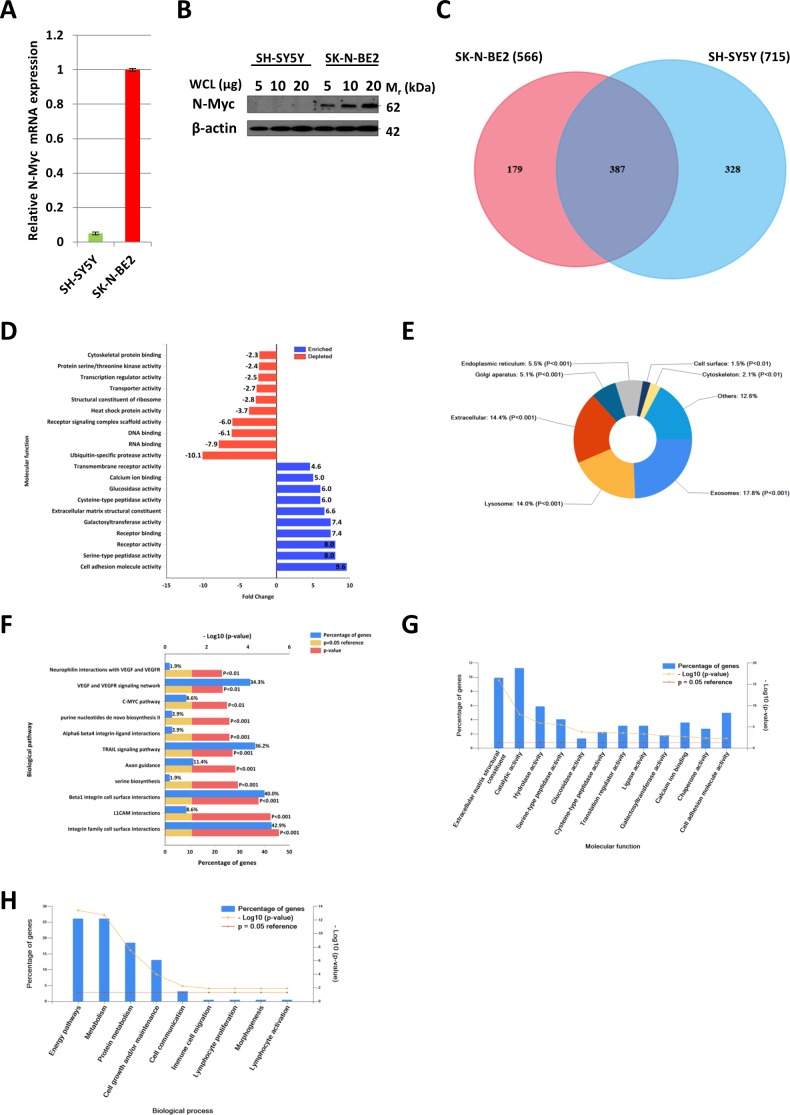
Comparison of N-Myc amplified and non-amplified neuroblastoma cell line secretome using FunRich (A) Relative mRNA expression of N-Myc against the housekeeper gene, hUBC, was determined by qPCR. Error bars represent standard error of the mean, n=3. (B) Western blotting confirmed the presence of N-Myc in SK-N-BE2, while absent in SH-SY5Y. β-actin was used as a loading control. (C) Venn diagram illustrating comparison of proteins detected in N-Myc amplified SK-N-BE2 and non-amplified SH-SY5Y secretome. The numbers outside represent the total number of proteins identified in the respective sample. (D) Enrichment and depletion of molecular functions in the secretome of SK-N-BE2 compared to SH-SY5Y is depicted. (E) Donought graph of subcellular localization of highly abundant proteins in SK-N-BE2 secretome (F) Column graph of biological pathways overrepresented in highly abundant proteins in SK-N-BE2 secretome (G) Bar graph of molecular function overrepresented in highly abundant proteins in SK-N-BE2 secretome is depicted (H) Bar graph of biological processes overrepresented in highly abundant proteins in SK-N-BE2 secretome is shown.

Quantitative PCR (Figure 1A) and Western blot analysis (Figure 1B) of SH-SY5Y and SK-N-BE2 cells confirmed the overexpression of N-Myc in SK-N-BE2 cells. To identify the secreted proteins, a proteomic analysis was performed on the secretome of N-Myc amplified (SK-N-BE2) and non-amplified (SH-SY5Y) neuroblastoma cells. Cells were grown in serum-free media for 24 h and the conditioned media was collected, concentrated, and subjected to in-gel digestion and mass spectrometry-based label free quantitative proteomic analysis. Trypan blue-based cell death assay revealed more than 8.5% and 7.8% cell death in SH-SY5Y and SK-N-BE2 cells (Supplementary Figure 1). Overall, 894 secreted proteins were detected (Supplementary Table 1). Among these, 328 and 179 proteins were unique to the SH-SY5Y and SK-N-BE2 secretome, respectively (Figure 1C). A total of 387 proteins were common between the cells, among which 49 and 90 proteins were of higher and lower abundance, respectively, in SK-N-BE2 compared to SH-SY5Y. In order to identify proteins that are overrepresented in any functional class, the dysregulated proteins were subjected to functional enrichment analysis using FunRich software (Figure 1D-H). According to the molecular functions, proteins more abundant in the N-Myc amplified SK-N-BE2 secretome were enriched (compared to non-N-Myc amplified SH-SY5Y) in cell adhesion (9.6-fold), cysteine-type peptidase activity (6-fold), serine-type peptidase activity (8-fold), galactosyltransferase activity (7.4-fold), ECM structural constituent (6.6-fold) and receptor binding (7.4-fold) (Figure 1D). By contrast, proteins identified in SK-N-BE2 secretome were depleted of RNA and DNA binding proteins (7.9- and 6.1-fold, respectively), ubiquitin specific protease activity (10-fold), and transporter activity (2.7-fold). The increase in cell death (~1%) in SH-SY5Y cells could have also contributed to the identification of DNA/RNA binding proteins in the secretome of SH-SY5Y cells.

Further to this, 14% of proteins more abundant in SK-N-BE2 secretome were categorised as extracellular (Figure 1E). More than 17.8% of the proteins were identified in exosomes, a class of extracellular vesicles [20]. Among the pathways, integrin family cell surface interactions, beta1 integrin cell surface interactions, L1CAM interactions, TRAIL signaling pathway, C-Myc pathway and Aplha6 beta4 integrin-ligand interactions were significantly overrepresented in proteins more abundant in the SK-N-BE2 secretome (Figure 1F). Proteins implicated in ECM structure, catalysis, hydrolysis and serine peptidase activity were significantly overrepresented in SK-N-BE2 secretome (Figure 1G). Extracellular matrix components biglycan (BGN) and Collagen, type VI, alpha 1II (COL6A3) were uniquely found in SK-N-BE2 secretome. Other ECM components that were highly abundant and unique to SK-N-BE2 secretome include collagens and proteoglycans such as COL6A2 (26-fold), HSPG2 (22-fold), COL14A1 (11-fold), LUM (9-fold), DCN (7-fold), COL21A1 (5-fold). In the context of biological processes, proteins involved in energy pathways, metabolism, cell growth and/or maintenance and cell communication were enriched in SK-N-BE2 secretome (Figure 1H).

When only the proteins common to both secretomes were analysed, extracellular matrix constituents and cell adhesion molecules were overrepresented. The extracellular matrix is not a passive structure which solely acts as a scaffold for the movement of neural crest cells. Rather, it provides an active input for cell migration from the neural tube. In this matrix, fibronectin, laminins, and proteoglycans regulate cell movement [21]. Proteins involved in laminin-10/11 complexes were also among the enriched proteins in SK-N-BE2 secretome (LAMC1 by 6-fold and LAMA5 by 3-fold). It is established that Laminin 10/11 complex is involved in neoplasia by promoting proliferation and migration [22]. The higher abundance of proteins that facilitate cell proliferation, migration and invasion in SK-N-BE2 secretome highlights the role of these proteins in the aggressiveness and invasiveness of the N-Myc amplified neuroblastoma cells.

There were 28 proteins, mostly ECM constituents (i.e. BGN, COL6A3, COL6A2, HSPG2 and THBS2), that showed more than 15-fold increase (arbitrary cut-off) in SK-N-BE2 secretome compared to SH-SY5Y. The transmembrane glycoprotein teneurin-4 (ODZ4), which is a highly expressed cellular signal transducer in neurons, was the one of the most highly abundant proteins in SK-N-BE2 secretome. ODZ4 is a positive regulator of myelination in the CNS and promotes activation of focal adhesion kinase. Interestingly, among the 28 proteins, there were several cathepsin family members.

### SK-N-BE2 and SH-SY5Y secretome are enriched in neuroblastoma biomarkers

In addition to providing functional clues on neuroblastoma pathogenicity, the secretome protein fraction may also contain potential biomarkers which could be exploited as indicators of the disease condition. Both SK-N-BE2 and SH-SY5Y secretome contained proteins that were previously implicated in neuroblastoma pathogenesis. Granin family members such as secretogranin II (SgII), chromogranin B (SgI) and VGF nerve growth factor inducible (VGF) are known biomarkers of neuroblastoma [23]. Even though VGF and SgI were common to both secretomes, they were enriched by 50- and 6-fold, respectively, in SK-N-BE2 cells. SgII was exclusively identified in SK-N-BE2 secretome (141-fold). The observation is consistent with previous findings that reported high SgII expression levels in SK-N-BE2 cells compared to SH-SY5Y [24]. In addition, SgII has also been suggested as a marker of human neuroblastoma cell differentiation [25]. L1 cell adhesion molecule (L1CAM) was the fourth most abundant protein in SK-N-BE2 (72-fold highly abundant compared to SH-SY5Y) and is known to be an indicator for developing neuronal cells in more mature stages of neuroblastoma [26]. The motility, angiogenesis and metastasis-stimulating factor Autotaxin (ENPP2) [27], was 65-fold enriched in SK-N-BE2. Similarly, dopa decarboxylase (DDC) was 28-fold highly abundant in SK-N-BE2. High expression of DDC both in blood and marrow corresponds to metastatic neuroblastoma at diagnosis, residual disease, and poor outcome [28].

### EMT markers are enriched in SK-N-BE2 secretome

**Figure 2:**
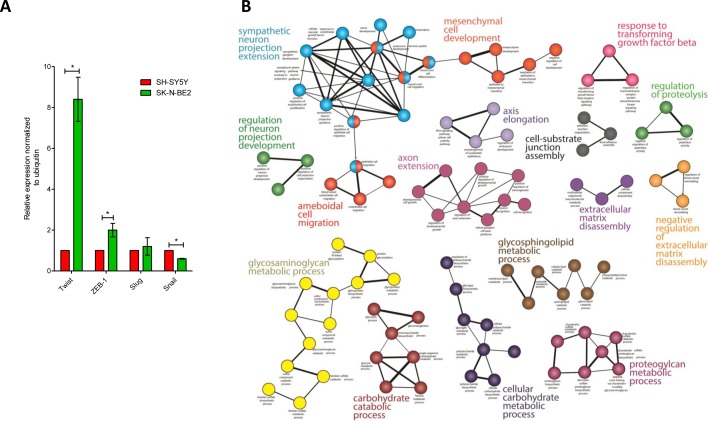

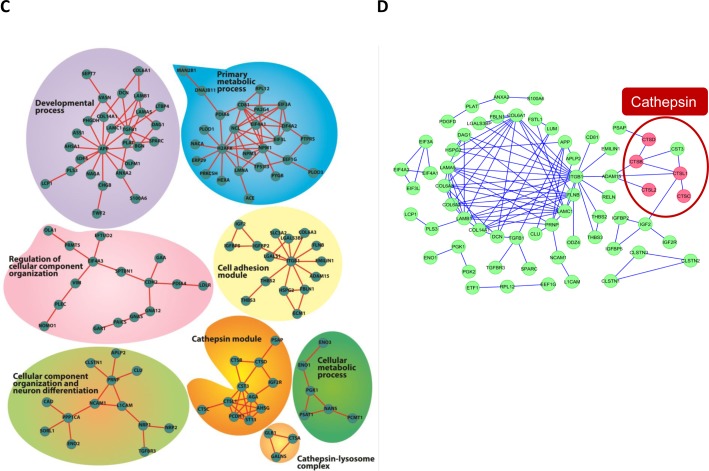
EMT regulators and functional network analysis of neuroblastoma cells and secretome (A) Relative mRNA expression of the four transcription factors that govern EMT is depicted based on qPCR analysis. Error bars represent standard error of the mean. n=3, * denotes significance (p<0.05). Student's *t*-test was used to evaluate statistical significant differences between the values. (B) The functional networks obtained by ClueGO, a Cytoscape plugin, depicts the presence of highly significant leading functional groups. (C) ClusterMaker-based modules that are highly clustered are depicted in the figure. (D) Visualization of protein interaction networks using Reactome plugin in Cytoscape. Cathepsin proteases are enriched and form a sub-network.

Epithelial to Mesenchymal Transition (EMT) is a mechanism by which cells lose their epithelial characteristics and acquire more migratory mesenchymal properties [29]. EMT is an important mechanism for both the initiation of tumour invasion and subsequent metastasis of neuroblastoma [30]. Proteomic analysis highlighted the abundance of EMT markers such as Vimentin and SPARC (33- and 14-fold, respectively) in the more aggressive SK-N-BE2 neuroblastoma cell-derived secretome. Similarly, N-Cadherin was 3-fold more abundant in SK-N-BE2 secretome. In addition, proteins attributed to the mesenchymal transition signature (THBS2 (21-fold), BGN (312-fold), LGALS1 (5-fold), LUM (8-fold), SERPINF1 (12-fold)) were also more abundant in SK-N-BE2 secretome [31]. MMP2 was a common mesenchymal transition signature protein that was present in almost equal amounts in both secretomes. MMP2 is an important protein in the degradation of various extracellular matrix molecules, including collagen type I, IV, V, VII, X, and XI, as well as elastin, gelatins and laminin [32]. To further validate the EMT phenotype, qPCR analysis was performed for EMT regulators Snail, Slug, Twist and ZEB-1. As shown in Figure 2A, Twist was 8.5-fold upregulated in SK-N-BE2 cells compared to SH-SY5Y. Similarly, ZEB-1 was 2-fold upregulated in SK-N-BE2 cells correlating with the EMT phenotype. However, Slug did not show any significant difference in the abundance among the two neuroblastoma cells. In addition, Snail was significantly downregulated in SK-N-BE2 cells. These observations clearly highlight that EMT may also contribute to neuroblastoma aggressiveness.

### Several N-Myc targets are dysregulated in SK-N-BE2 secretome

Overexpression of N-Myc in SK-N-SH neuroblastoma cells lacking N-Myc resulted in the marked increase of insulin like growth factor (IGF)-2, IGF-1R and IGF-binding protein (IGFBP)-2 suggesting transcriptional regulation of these proteins by N-Myc [33]. Consistent with this finding, N-Myc amplified SK-N-BE2 cells secreted IGF-2 (5-fold) and IGFBP-2 (4-fold) in higher abundance compared to SH-SY5Y cells. However, IGF-1R was not detected in SK-N-BE2 secretomes, though a 4-fold increase in shed IGF-2R domains in the N-Myc amplified SK-N-BE2 secretome was observed. It is well established that IGF stimulate proliferation and differentiation in many cell types and are important in the progression of neuroblastoma [33]. Additionally, other known N-Myc targets such as Cathepsin D and N-CAD [34, 35] were also highly abundant in SK-N-BE2 secretome.

Dihydropyrimidinase-like proteins (DPYSLs) are a family of proteins developmentally regulated during maturation of the nervous system. It has been previously established that DPYSLs are negatively regulated by N-Myc and reduced expression levels correlate with poor clinical outcomes [36]. In accordance with this observation, DPYSL1, 2, 4 and 5 were less abundant in SK-N-BE2 secretome. DPYSL1, 2, 4 and 5 exhibited 3-, 4-, 12- and 10-fold less abundance in SK-N-BE2 secretome, respectively.

### Cathepsins are enriched in N-Myc amplified SK-N-BE2 cell-derived secretome

To gain functional insights and to depict the biological modules in highly abundant SK-N-BE2 proteins, a functional interaction network analysis approach using ClueGO cytoscape plugin was performed. The functional networks (Figure 2B) depict the presence of highly significant functional groups known to be involved in many developmental and basic metabolic process like the development of the nervous system, mesenchymal cell, and neuron projections, axon extension, axis elongation, extracellular matrix disassembly and glycosaminoglycan metabolism. Further, the protein-protein interaction network analysis of these highly abundant proteins depicts the presence of highly clustered cathepsin modules as well as other biological modules, which are known to play a role in the developmental and metabolic process (Figure 2C).

Additionally, using Reactome Plugin in Cytoscape, a protein interaction network analysis was performed with the highly abundant proteins in the SK-N-BE2 secretome. This analysis highlighted the enrichment of the cathepsin protein network in SK-N-BE2 secretome (Figure 2D). Proteases of the cathepsin family cleave amide bonds of peptides and proteins. While cathepsins are predominantly contained within lysosomes and are active at acidic pH, they may also be associated with the plasma membrane or secreted into the extracellular environment [37]. Cathepsins can be divided into three subgroups according to their active-site amino acid; i.e. cysteine cathepsins (B, C, H, F, K, L, O, S, V, W and Z/X), aspartic cathepsins (D and E) or serine cathepsins (A, G). Among the 228 proteins with at least two-fold increase in SK-N-BE2 secretome, 6 were cathepsins, including CTSL1 (29-fold), CTSD (27-fold), CTSB (20-fold), CTSC (9-fold), CTSA (6-fold) and CTSL2 (3-fold).

**Figure 3:**
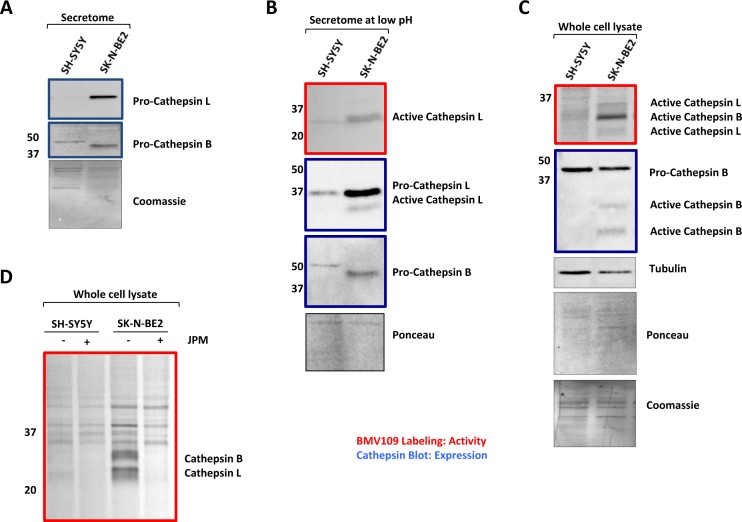
Cathepsin expression and activity of secretomes and cells (A) Western blot analysis for cathepsin abundance in the neuroblastoma secretome samples. Higher abundance of pro-cathepsins B and L is detected in the secretome of SK-N-BE2 compared to SH-SY5Y (B) BMV109 probe based cathepsin activity analysis in secretomes. Under low pH conditions (5.5), the active form of cathepsin L is present in higher abundance in the secretome of SK-N-BE2 compared to SH-SY5Y. (C) Probe based cathepsin activity and expression analysis in whole cell lysates. SK-N-BE2 whole cell lysates exhibit higher levels of active cathepsins B and L as compared to SH-SY5Y whole cell lysates. (D) Cathepsin activity in the whole cell lysates in the presence and absence of cathepsin inhibitor JPM. JPM treatment leads to ablation of cathepsin activity in SK-N-BE2 cells.

Enhanced secretion of cathepsins in SK-N-BE2 secretome was further validated biochemically (Figure 3). Consistent with the proteomic data, Western blotting confirmed the secretion of high amounts of pro-cathepsin L by SK-N-BE2 cells (Figure 3A). Cathepsin B was also present in its proform in both cell lines; however, SK-N-BE2 cells secreted a lower molecular weight species, which may be the result of increased autocatalytic maturation or processing by another protease. To assess levels of *active* cathepsins, the pH of the conditioned media was lowered to 5.5 to allow for optimal activation and an activity-based probe, BMV109, was added followed by SDS-PAGE and fluorescence detection. This assay revealed increased activity of cathepsin L in the secretome of SK-N-BE2 compared to SH-SY5Y cells (Figure 3B). Subsequent Western blotting revealed processing of a small amount of cathepsin L to its active form, which was not observed for cathepsin B. This data suggests that within a tumour microenvironment *in vivo*, where hypoxia and other factors are known to create an acidic extracellular environment, SK-N-BE2 cells are likely to secrete cathepsin L capable of proteolytic activity.

Furthermore, the activity and expression of cathepsins in whole cell lysates from the two neuroblastoma cells were also analysed (Figure 3C). Expression of the proform of cathepsin B was relatively similar between the two whole cell lysates; however, the SK-N-BE2 line displayed two more mature cathepsin B species, which correspond to the activated forms. Expression levels of procathepsin L could not be detected in the whole cell lysates by Western as the expression levels were below the threshold of detection. Activity of cathepsins B and L, as assessed by BMV109 labelling, was increased in SK-N-BE2 compared to SH-SY5Y cells (Figure 3C). Cathepsin activity could be abolished by treating the cells with the cysteine cathepsin inhibitor JPM-OEt prior to adding BMV109 (Figure 3D). As cathepsins are known to facilitate cancer progression by protein degradation and ECM remodelling [38], the higher abundance of active cathepsins in SK-N-BE2 secretome may contribute to cancer aggressiveness and chemotherapeutic sensitivity in N-Myc amplified SK-N-BE2 cells.

### Cathepsins inhibition sensitizes neuroblastoma cells to doxorubicin

**Figure 4:**
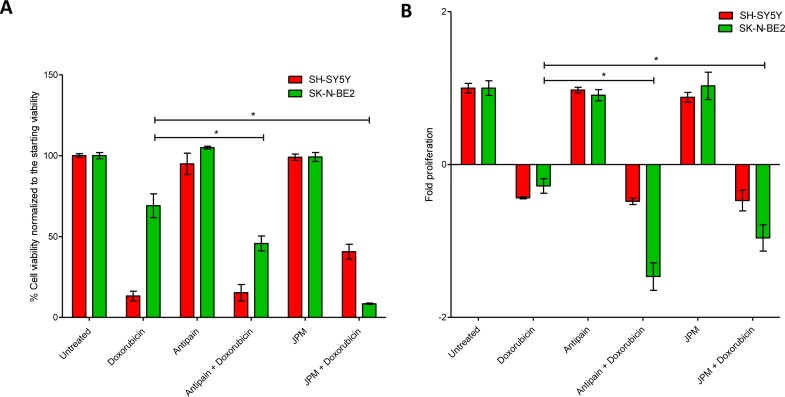
Treatment of neuroblastoma cells with cathepsin inhibitors and doxorubicin (A) Cell viability analysed with propidium iodide uptake and annexin V-FITC staining. Error bars represent standard error of the mean, n=3, * denotes significance (p<0.05). (B) Cell proliferation analysis of neuroblastoma cells treated with doxorubicin in the presence and absence of cathepsin inhibitors. Error bars represent standard error of the mean, n=12, * denotes significance (p<0.05). Student's *t*-test was used to evaluate statistically significant differences between the values.

Chemotherapeutic resistance is the primary cause of treatment failure in N-Myc amplified neuroblastoma patients [16, 17]. It has been reported that cellular cathepsin D play a role in regulating chemotherapeutic drug resistance in N-Myc amplified Tet21N neuroblastoma cells [35]. As cathepsins were highly abundant in the secretome of N-Myc amplified neuroblastoma cells, the role of cathepsins in regulating sensitivity to a chemotherapeutic drug was studied. In order to do this, N-Myc amplified SK-N-BE2 cells were co-treated with doxorubicin and a cathepsin inhibitor. Two cathepsin inhibitors namely JPM-OEt, a cysteine cathepsin inhibitor [39] and antipain dihydrochloride, a reversible cysteine, aspartic and serine cathepsin inhibitor [40], were used in this analysis. FACS and MTS assays were performed to monitor the cell death and proliferation upon treatment with doxorubicin (Figure 4). Consistent with previous reports, SK-N-BE2 cells were more resistant to doxorubicin treatment compared to SH-SY5Y (Figure 4A) [16, 17]. Cell death assay with propidium iodide uptake and Annexin V staining was performed with the two neuroblastoma cell lines treated with doxorubicin in the presence and absence of cathepsin inhibition. SH-SY5Y did not show a significant difference in the level of cell death when co-treated with doxorubicin and antipain. However, SK-N-BE2 cells exhibited higher levels of apoptosis when co-treated with doxorubicin and antipain or JPM (Figure 4A). Cysteine cathepsin inhibitor JPM co-treatment with doxorubicin induced more cell death in SK-N-BE2 cells than antipain. Importantly, for the first time, this data suggests that that cysteine cathepsins B, L and C may protect the neuroblastoma cells from doxorubicin induced cell death.

In addition, for unknown reasons, the cell viability was higher in SH-SY5Y cells when co-treated with JPM and doxorubicin compared to doxorubicin alone. However, this trend was not visible in MTS based proliferation assay (Figure 4B). As MTS and the FACS assays profile for two different readouts, it is not uncommon to obtain varying results. On the contrast, consistent with FACS apoptosis assay, a significant difference in cell proliferation and metabolic flux was observed for SK-N-BE2 cells treated with doxorubicin in the presence of cathepsin inhibitors compared to doxorubicin treatment alone (Figure 4B). The level of cell proliferation was almost the same for more drug responsive SH-SY5Y both in the presence and absence of cathepsin inhibitors with doxorubicin treatment. These results confirm that cathepsin inhibition could sensitize the aggressive N-Myc amplified neuroblastoma cells to doxorubicin.

### Cathepsins inhibition affect the migratory properties of SK-N-BE2 cells more than SH-SY5Y

**Figure 5:**
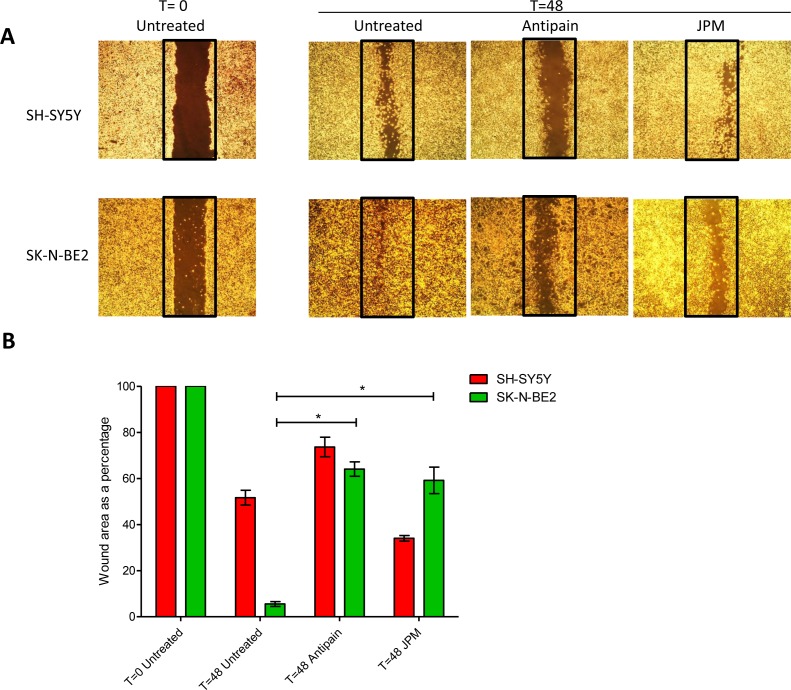
Role of cathepsins in migratory properties of N-Myc amplified and non-amplified neuroblastoma cells (A) Wound healing assay of neuroblastoma cell lines SK-N-BE2 and SH-SY5Y. Wound was performed after confluence, and cells were treated with either one of the cathepsin inhibitors. Migration was assessed at 48 h after wounding. Images were taken under the 4x objective of the light microscope. The images are representative of three independent experiments. (B) Quantification of wound closure. Error bars represent standard error of the mean, n=3, * denotes significance (p<0.05). Student's *t*-test was used to evaluate statistically significant differences between the values.

In addition to chemosensitivity, the role of cathepsins in cell migration was studied by wound healing assay. Wound healing assays were performed by making a uniform scratch on a monolayer of cells at 100% confluence. The scratches were then observed at different time points for closure of the wound gap (Figure 5). It was observed that the wound closure rate was faster in N-Myc amplified neuroblastoma cells confirming the aggressive phenotype of the cells (Figure 5A). When wound healing assay was performed with the presence of cathepsin inhibitors, the migratory properties of SK-N-BE2 cells were significantly attenuated in comparison to SH-SY5Y cells (Figure 5B). The two cathepsin inhibitors reduced the migration to a similar extent. These data suggest that cathepsins indeed play a role in migration of the SK-N-BE2 cells.

Overall, a total of 894 proteins were identified in the secretome-based proteomics analysis of the highly aggressive N-Myc amplified SK-N-BE2 and less aggressive N-Myc non-amplified SH-SY5Y neuroblastoma cells. A following bioinformatics analysis of the secretome data showed multiple classes of proteins that are overrepresented in SK-N-BE2 secretome. Among these, the most significant findings include the involvement of EMT and the enrichment of the protease cathepsins. EMT results in the loss of polarity and the gain of more invasive phenotype which is consistent with the SK-N-BE2 cells. The mesenchymal attributes thereby could be one of the main reasons for the aggressive nature of SK-N-BE2 cells.

In addition to metastasis, recent evidences suggest that EMT also regulates chemotherapeutic drug resistance in a number of cancer types. Human colorectal cancer cell lines KM12L4 and HT29 displayed EMT as a consequence of oxiplatin resistance by translocating β-Catenin to the nucleus [41]. Hepatocellular carcinoma cells resistant to 5-FU also showed induction of EMT with the upregulation of Twist and the down regulation of E-Cadherin [42]. Similarly, activation of Snail in colorectal cancer cell lines led to increased motility and invasiveness with increased resistance to 5-FU [43]. With the light of these observations, it can be speculated that EMT may also contribute to the inherent treatment resistance of SK-N-BE2 cells.

The most significant findings of the study relate to cathepsins. For the first time, the secretion of active cathepsins by neuroblastoma cells is shown in this study. It is well documented that cathepsins are upregulated in a variety of cancers resulting in the secretion of both inactive and active forms during cancer progression [44]. Secreted cathepsins can function in proteolytic pathways thereby increasing cancer aggressiveness. Mass spectrometry-based proteomics analysis highlighted the secretion of more than six cathepsins by SK-N-BE2 cells. Most importantly, the detection of active cathepsins in the secretome suggests the involvement of these proteases in the tumor microenvironment and in turn in N-Myc-based neuroblastoma aggressiveness. FACS and MTS based assays confirmed the involvement of the cathepsins in treatment resistance to doxorubicin. As N-Myc amplified neuroblastoma is inherently resistant to doxorubicin, cathepsin inhibition-based sensitization of N-Myc amplified neuroblastoma cells provides unparalleled opportunities to treat neuroblastoma patients effectively.

## CONCLUSIONS

Secretome profiles of N-Myc amplified (SK-N-BE2) and non-amplified (SH-SY5Y) neuroblastoma cells were identified by high-resolution mass spectrometry-based label-free quantitative proteomics. Functional enrichment analysis of identified proteins revealed multiple attributes that are overrepresented in SK-N-BE2 secretome. The protein-protein interaction-based network analysis identified the enrichment of EMT as well as the cathepsin sub-networks. Inhibition of cathepsins increased the sensitivity of SK-N-BE2 cells to doxorubicin and attenuated its migratory properties. However non-amplified SH-SY5Y cell line remained unaffected or mildly affected by cathepsin inhibition. These data indicate the prominent role of cathepsins in the N-Myc amplified neuroblastoma pathogenesis. As N-Myc amplification correlates with aggressive neuroblastoma and chemotherapy-based treatment failure, co-treatment with cathepsin inhibitors will be a better avenue for disease management.

## MATERIALS AND METHODS

### Cell culture

Human neuroblastoma cell lines SH-SY5Y and SK-N-BE2 were gifted by Dr. Julie Atkins (Department of Biochemistry, La Trobe University) and Dr. Loretta Lau (Sydney Medical School, University of Sydney). Both the cell lines were grown in DMEM (GIBCO, Life Technologies) containing 10% fetal calf serum (FCS) and 100 Unit/mL of penicillin-streptomycin (GIBCO, Life Technologies). The cells were cultured in 5% CO_2_ atmosphere at 37°C.

### Preparation of conditioned media (CM)

For preparing CM, neuroblastoma cells were seeded in 150 mm diameter culture dishes (10 dishes per cell line) in the presence of 25 mL of culture medium. After cell density reached 70-80% confluence, the cells were washed twice with phosphate buffered saline (PBS). The cells were then cultured in 15 mL of DMEM supplemented with 0.8% Insulin transferrin selenium (ITS) and 100 Unit/mL of penicillin-streptomycin for 24 h. The CM was collected, centrifuged at 500 g for 10 min to remove floating cells followed by another centrifugation step at 2,000 g for 20 min. The CM was then centrifuged for 1 h at 100,000 g to remove extracellular vesicles. The CM was concentrated from 50 mL to ~1 mL using an Amicon Ultra-15 centrifugal filter device with 3,000 Da nominal molecular weight limit (Millipore) at 4,000 g. The resulted concentrated CM was stored at −80°C until further analysis.

### In gel digestion

Concentrated CM (30 μg quantified by SYPRO Ruby staining) was electrophoretically separated using SDS-PAGE and proteins were visualized by staining with Coomassie Brilliant Blue stain. Gel lanes were cut into 20 × 2 mm bands using a scalpel blade and proteins were reduced, alkylated and trypsinised as described previously [45]. Briefly, the gel bands were subjected to reduction by 10 mM DTT (Bio-Rad), alkylation by 25 mM iodoacetamide (Sigma), tryptic digestion overnight with 150 ng of trypsin (Promega). Subsequently, the tryptic peptides were further extracted using acetonitrile (50% w/v) and 0.1% trifluoroacetic acid (0.1%).

### LC-MS/MS

Extracted tryptic peptides from each gel band were concentrated to ~10 μL by centrifugal lyophilisation and analysed by LC-MS/MS using LTQ Orbitrap Velos mass spectrometer (Thermo Scientific) fitted with nanoflow reversed-phase-HPLC (Model 1200, Agilent). The nano-LC system was equipped with an Acclaim Pepmap nano-trap column (Dionex – C18, 100 Å, 75 μm × 2 cm) and an Acclaim Pepmap RSLC analytical column (Dionex – C18, 100 Å, 75 μm × 15 cm). Typically for each LC-MS/MS experiment, 1 μL of the peptide mix was loaded onto the enrichment (trap) column at an isocratic flow of 3 μL/min of 3% acetonitrile containing 0.1% formic acid for 4 min before the enrichment column is switched in-line with the analytical column. The eluents used for the LC were 0.1% v/v formic acid (solvent A) and 100% acetonitrile/0.1% formic acid v/v. The gradient used was 3% B to 8% B for 1 min, 8% B to 35% B in 30 min, 35% B to 85% B in 5 min and maintained at 85% B for the final 5 min. All spectra were acquired in positive mode with full scan MS spectra scanning from m/z 300–2000 in the FT mode at 30000 resolution after accumulating to a target value of 1.00e6 with maximum accumulation of 500 ms. The 20 most intense peptide ions with charge states ≥2 were isolated at a target value of 1000 and fragmented by low energy CID with normalized collision energy of 30 and activation Q of 0.25. Dynamic exclusion settings of 2 repeat counts over 30 s and exclusion duration of 70 s.

### Database searching and protein identification

Peak lists were generated using extract-msn as part of Bioworks 3.3.1 (Thermo Scientific) using the following parameters: minimum mass 300; maximum mass 5,000; grouping tolerance 0.01 Da; intermediate scans 200; minimum group count 1; 10 peaks minimum and total ion current of 100. Peak lists for each LC-MS/MS run were merged into a single mascot generic format. Automatic charge state recognition was used because of the high resolution survey scan (30,000). LC-MS/MS spectra were searched against the NCBI RefSeq database [46] in a target decoy fashion using MASCOT (v2.4, Matrix Science, U.K.). Search parameters used were: fixed modification (carboamidomethylation of cysteine; +57 Da), variable modifications (oxidation of methionine; +16 Da), three missed tryptic cleavages, 20 ppm peptide mass tolerance and 0.6 Da fragment ion mass tolerance. Peptide identifications with mascot ion score greater than the identity score were deemed significant. With a cut-off of two unique peptides per protein, less than 1% false discovery rate was achieved.

### Label-free spectral counting

The relative protein abundance between the samples was obtained by estimating the ratio of normalized spectral counts (RSc) as previously described [45].

RSc for protein A = [(*sY+c*) (*TX-sX+c*) / (*sX+c*) (*TY-sY+c*)]

Where *s* is the significant MS/MS spectra for protein A, *T* is the total number of significant MS/MS spectra in the secretome sample, *c* is the correction factor set to 1.25, and *X* and *Y* are the secretome samples. When RSc is less than 1, the negative inverse RSc value was used.

### RNA isolation

RNA isolation was performed using TRI reagent® RT (Molecular research, Inc). The cells were grown to 100% confluence and the medium was removed before adding 1 mL of TRI reagent. Repetitive pipetting was performed to obtain homogenized mixture of cells. The cell lysate was then aliquoted along with 50 μL of 4-bromoanisole (BAN) solution (Molecular research, Inc) and was subjected to vigorous mixing. To achieve phase separation, samples were subjected to centrifugation at 12,000 g for 5 min at 4°C. The top aqueous layer was separated out and equal volume of isopropanol was added. The mixture was then incubated for 10 min at room temperature. RNA pellet was obtained by centrifugation at 12,000 g for 5 min at 4°C. Ethanol (75% (v/v)) was used to wash the RNA pellet, which was then subjected to centrifugation. The pellet obtained was resuspended in Ambion® DEPC-treated water (Life technologies) and stored at −20°C.

### cDNA synthesis and qPCR

iScript™ cDNA synthesis kit (Bio-Rad) was used in synthesizing cDNA, according to manufacturer's protocol. Total RNA (2 μg) was used in the cDNA synthesis with 500 ng/uL as the final concentration of the reaction. The concentrations of the generated cDNA were measured using NanoDrop® ND-1000 (Thermo scientific) spectrophotometer. According to manufacturer's instructions, quantitative PCR was carried out using SensiMix™ SYBR Low-ROX kit (Bioline). For each reaction, appropriate primers were used. Activation of polymerase was carried out by heating the final qPCR mixture at 95°C for 10 min, followed by 40 cycles of amplification at 95°C for 15 sec, 52°C for 15 sec and 72°C for 15 sec using Agilent LC140 qPCR machine. The qPCR results obtained (with the use of generated cDNA) were normalized using the Ct values of human ubiquitin.

### Functional enrichment and interaction network analysis

The functional networks of identified proteins was constructed using ClueGO v1.7.1 [47], a Cytoscape v2.8.3 [48] plugin. Overrepresentation of Gene Ontology biological process and pathway terms for N-Myc amplified and non-amplified highly abundant proteins were identified using the stand-alone enrichment analysis tool FunRich. The protein-protein physical interactions for the highly abundant N-Myc amplified genes were collated from HPRD [49] and BioGRID [50] interaction databases and the interaction networks were visualized using Cytoscape v.2.8.3. The protein-protein interaction networks were further separated into different clusters and biological significance of these clusters were depicted using clusterMaker v.1.8 and BiNGO v.2.44 cytoscape plugins, respectively.

### Cathepsin analysis in the secretome and whole cell lysates

SH-SY5Y and SK-N-BE2 neuroblastoma cells were seeded in equal density in 6-well plates. When cells reached 80% confluency, 0.1 μM BMV109 pan cathepsin activity-based probe [51] was added to the cell and incubated for 1 h. Following incubation cells were washed thrice with PBS. Cells were then harvested by scraping and lysed for analysis. Lysis buffer consisted of 50 mM citrate, pH 5.5, 0.5% CHAPS, 0.1% Triton X-100 and 4 mM DTT. The lysates were quantified using BCA assay and equal amount of sample (30 μg) was analysed on a gel. For cathepsin analysis, 30 μg of total protein from each secretome sample was taken and BMV109 probe (0.1 μM) was added to each sample and incubated at 37°C for 1 h. At the end of the incubation, SDS sample loading buffer was added to each sample and analysed on a gel. The Cy5 fluorescence of the probe was detected by scanning the gels under the Typhoon Trio (GE Healthcare) scanner. The gels was then transferred to a nitrocellulose membrane and probed with antibodies against Cathepsin isoforms B and L (R&D AF1515 and AF965 antibodies, respectively). The specificity of the probe was tested by growing cells in the presence of the cysteine cathepsin inhibitor JPM (100 μM) for 24 h and then incubating with the probe.

### Doxorubicin mediated cell death assay and proliferation assay

Equal numbers of cells were seeded in 24-well plates (for cell death analysis) and 96-well plates (for proliferation analysis). After 12 h, cells were treated with cathepsin inhibitors (100 μM cysteine cathepsin inhibitor JPM (Drug Synthesis and Chemistry Branch, Division of Cancer Treatment and Diagnosis, National Cancer Institute, MD), 250 μM antipain dihydrochloride (Santa Cruz Biotech) or 10 μg/mL cathepsin inhibitor peptide (Santa Cruz Biotech). After 24 h of the addition of cathepsin inhibitors (t=0), cells were treated with 1 μM doxorubicin (Hospira Inc). Post doxorubicin treatment at 48 h (t=48), cell viability was measured by propidium iodide uptake and annexinV-FITC staining by FACS. The proliferation was detected by MTS assay at 0 h (t=0) and 48 h (t=48) time points. MTS solution (PMS reagent (Sigma Life Science®) in DPBS and CellTiter 96® AQuueous MTS reagent powder (Promega) in DPBS at the ratio of 1:20, according to manufacturer's protocol) was added to each well. The plate was incubated for 1.5 h after the addition of MTS solution. The absorbance wave lengths used were 490 nm and 630 nm and the plate was read using SpectraMaxM5 multi-mode microplate reader (Molecular Devices). For FACS analysis both the detached and attached cells were harvested and spun at 2000 *g*. The cell pellet was washed and stained with annexin V-FITC (Invitrogen, Molecular probes) in FACS buffer (150 mM NaCl, 3.7 mM KCl, 2.5 mM CaCl2, 1.2 mM MgSO4, 7.4 mM HEPES-NaOH, 1.2 mM KH2PO4, 0.8 mM K2HPO4) supplemented with 5% FCS) for 15 min according to the manufacturer's instructions. Following this step, the cells were centrifuged and washed once again in FACS buffer. The resulting cell pellet was resuspended in 50 μL of 2.5 ng/μL propidium iodide and the fluorescence was detected with BD FACS Canto.

### Wound healing assay

Equal numbers of cells were seeded in 6-well plates and were allowed to reach 100% confluence. A pipette tip was used to scratch the monolayer of cells. Detached cells were removed by changing the medium to fresh medium. Cells were then incubated at 37°C in 5% CO_2_ with and without cathepsin inhibitors. The width of the wound was monitored under the microscope at 0, 24 and 48 h.

### Statistical analysis

Statistical analysis was performed with Prism5 (GraphPad) and Microsoft Office Excel. All data shown are representative of results obtained from experiments conducted two or three times as specified in the specific sections. The results were analysed by T-tests.

## SUPPLEMENTARY MATERIAL, FIGURE AND TABLE


