# Supplementary material for: Inhibition of cathepsin proteases attenuates migration and sensitizes aggressive N-Myc amplified human neuroblastoma cells to doxorubicin
Source: Oncotarget. 2015 Mar 14;6(13):11175–90. doi: 10.18632/oncotarget.3579 (PMC4484448; doi:10.18632/oncotarget.3579)
Supplement: Supplementary file 1 [file oncotarget-06-11175-s001.pdf]

Inhibition of cathepsin proteases attenuates migration and sensitizes aggressive N-Myc amplified human neuroblastoma cells to doxorubicin

Supplementary Material

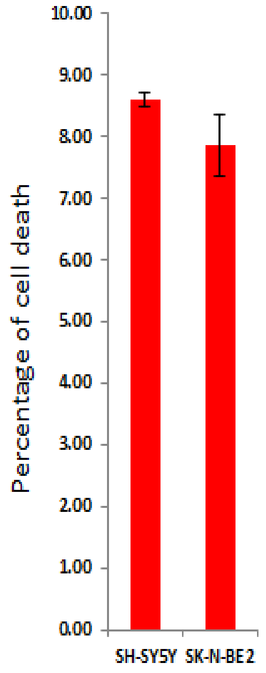

Supplementary Figure 1: Percentage of cell death in SH-SY5Y and SK-N-BE2 cells

Trypan blue assay was performed for assessing cell death in SH-SY5Y and SK-N-BE2 cells. The results depicted as histograms reveal the SH-SY5Y exhibited more than 8.5% cell death. Similarly, more than 7.8% cell death was observed in SK-N-BE2 cells. Error bars represent standard error of the mean, n=5.
